# Divergent Microbiota Dynamics along the Coastal Marine Ecosystem of Puerto Rico

**DOI:** 10.3390/microbiolres11020009

**Published:** 2020-12-21

**Authors:** Clifford Jaylen Louime, Frances Vazquez-Sanchez, Dieunel Derilus, Filipa Godoy-Vitorino

**Affiliations:** 1 Department of Environmental Sciences, University of Puerto Rico, San Juan, PR 00931, USA; 2 Department of Microbiology & Medical Zoology, School of Medicine, University of Puerto Rico, San Juan, PR 00936, USA

**Keywords:** bacterial diversity, marine ecosystems, 16S rRNA, NGS (Next Generation Sequencing), microbiome, Caribbean Sea, global climate change

## Abstract

Understanding the different factors shaping the spatial and temporal distribution of marine microorganisms is fundamental in predicting their responses to future environmental disturbances. There has been, however, little effort to characterize the microbial diversity including the microbiome dynamics among regions in the Caribbean Sea. Toward this end, this study was designed to gain some critical insights into microbial diversity within the coastal marine ecosystem off the coast of Puerto Rico. Using Illumina MiSeq, the V4 region of the 16S rRNA gene was sequenced with the goal of characterizing the microbial diversity representative of different coastal sites around the island of Puerto Rico. This study provided valuable insights in terms of the local bacterial taxonomic abundance, α and β diversity, and the environmental factors shaping microbial community composition and structure. The most dominant phyla across all 11 sampling sites were the Proteobacteria, Bacteroidetes, and Planctomycetes, while the least dominant taxonomic groups were the NKB19, Tenericutes, OP3, Lentisphaerae, and SAR406. The geographical area (Caribbean and Atlantic seas) and salinity gradients were the main drivers shaping the marine microbial community around the island. Despite stable physical and chemical features of the different sites, a highly dynamic microbiome was observed. This highlights Caribbean waters as one of the richest marine sources for a microbial biodiversity hotspot. The data presented here provide a basis for further temporal evaluations aiming at deciphering microbial taxonomic diversity around the island, while determining how microbes adapt to changes in the climate.

## 1. Introduction

A key objective in microbial ecology is to understand the main factors affecting the spatial and temporal distributions of microbial taxa in the environment. This is fundamental in predicting the ecosystem response to upcoming environmental changes. Marine microbiota is highly dilute and differs according to temperature, geography, and other environmental factors [1], changing in composition with symbiotic association to marine animal systems, such as corals [2–6] or sponges [7–9] or even anthropogenic impact [10]. The communities conformed by these microorganisms, respond to changes in the environment, such as shifts in the temperature as a result of climate change and nutrient cycling [11,12]. Indeed, by controlling the cycling of elements essential for life on earth, microbes are the backbone of all our planet’s ecosystems. From nitrogen/carbon fixation to sulfur metabolism, the role of microbes on Earth is so immensely significant, that life on Earth as we know it, would not exist, if all microbes were to disappear [13].

Next Generation Sequencing (NGS) projects, such as the Global Ocean Sampling (GOS) expedition in the Sargasso Sea—one of the largest metagenomic studies so far, comes with twice the data size of the human genome, found 1.2 million genes, and inferred the presence of at least 1800 bacterial species [14–16]. The Tara Expeditions have also revealed that single-celled eukaryotes (protists) are critical players in global biogeochemical cycling of nutrients and energy in the oceans [17]. This confirms the role of microbes in the global biogeochemical cycles of carbon and nitrogen as already discussed and the underlying evidence that genomic and metabolic plasticity are the basis of microbial adaptation in marine ecosystems [15]. However, the marine environment, with its dispersion due to water currents has also an inherent complexity, in contrast to the static terrestrial environment. This limits the understanding of microbiome dynamics in oceans, and therefore, highlights the importance of localized studies that define core microbiota analyses in different geographical locations so that in the coming years, a better picture of the global microbial composition and structure is available. Furthermore, understanding the general mechanisms that shape the spatial distribution of bacterial taxa in specific marine microclimates, is a prerequisite to predict these ecosystems responses to eventual and future environmental changes.

The tropical island of Puerto Rico is part of a volcanic island platform, in the northernmost Atlantic side marked by higher wave energy and terrigenous sediments, while the Caribbean side is dominated by carbonate platforms with coral reefs and dominantly carbonate sediments [18]. These inherent coastal differences are also expected to result in different microbial community structures. Understanding the different environmental parameters that shape the distribution of costal microbiomes is crucial to predict and anticipate their response to the ongoing and accelerated environmental changes. In the present study, we aimed to compare the microbial community structure across coastal sites, across littoral Puerto Rico, and samples were grouped in the areas of the Caribbean and Atlantic seas, studied according to the dissolved oxygen, pH, temperature, and salinity gradients. The results of this work pointed out the relative importance of considering some key environmental variables in understanding tropical marine ecosystem biomarkers.

## 2. Materials and Methods

### 2.1. Sample Collection and Processing

**Figure 1.**
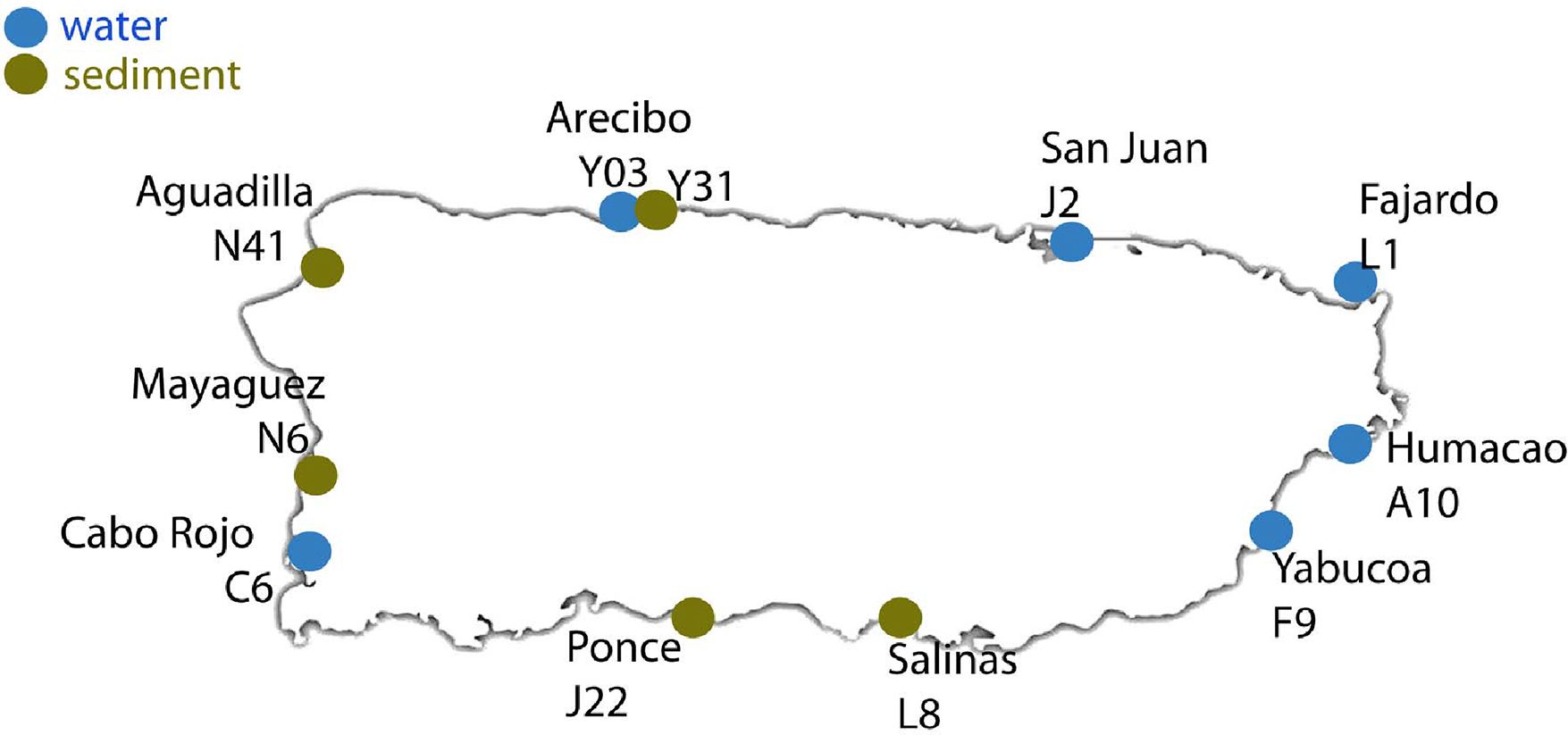
Diagram of the 11 sampling locations across the geography of Puerto Rico.

The collection and processing of seawater samples was performed according to a previous procedure [19]. During the month of May 2016, right at the transition of the dry and the rainy season on the island, a total of 11 samples, six seawater and five mostly from sediment, were taken at different sites across the geography of Puerto Rico including, the north, south east, and western coasts (Figure 1 and Table 1). For the meta-taxonomic analysis, replicate samples were drawn from the water at a depth between 3 and 10 m, using sterile 1-L polypropylene bottles and kept on ice until processing in the laboratory.

During the sampling expedition, various in situ physical measurements were performed on the water samples using the CTD probe (Fisher Scientific—Meter #6534251, Pittsburgh, PA, USA) equipped with water temperature, pH, salinity, and dissolved oxygen sensor. For genomic characterization, 1 L of each aggregated sample (water, sediment, and biofilms) was spun down at a moderate speed of 5000 rpm in order to avoid shearing of the DNA and the resulting pellet was stored frozen at −80 °C for later processing. Metagenomic DNA was isolated with the FastDNA^™^ Soil Kit (MPBiomedicals, Inc., Irvine, CA, USA), according to the manufacturer’s instructions. The quality and quantity of the DNA was first verified with the Nanodrop 1000 then run on a 1% DNA gel. The resulting purified DNA samples were stored at −80 °C for PCR analysis.

### 2.2. 16S rRNA Gene Amplicons and Sequence Analysis

The V4 hypervariable region of the 16S ribosomal RNA (~291 bp length) was amplified by PCR using the universal bacterial and archaeal primers: 515F (5′ GTGCCAGCMGCCGCGGTAA 3′) and 806R (5′ GGACTACHVGGGTWTCTAAT 3′) as used in the Earth Microbiome Project [20]. Amplicons of ~300 bp were multiplex paired-end sequenced in the Illumina Miseq platform. The resulting 16S rRNA gene sequences per sample were deposited in NCBI’s Bioproject PRJNA392167, ID: 392167 (NCBI 2018).

The software package QIIME v.1.9 (Quantitative Insights into Microbial Ecology) was used to analyze the generated sequences [21,22]. Processing was done as detailed [23]. Sequenced reads were demultiplexed and processed with a default Phred score of 20 (99% accuracy of the base calls), size (>200 bp), and chimera filtering with the Usearch Hierarchical Clustering Method [24].

Sequences were binned (97% homology) via the open reference-based method, implementing UCLUST [24] as the default OTU clustering tool used in QIIME’s workflows. Taxonomic classification was performed using the Ribosomal Database Project RDP v 2.2 [25] and the OTU table was built using the RDP previously assigned taxonomy and OTU map, which contains the OTU assignment and all of its sequences. The generated OTU table served as the basis for alpha diversity and beta diversity across sites. The same OTU table was used to statistically estimate the average abundance-based coverage of the samples. Finally, the taxa unique to each sample or shared among samples were analyzed and separated statistically using the R software package [26] to perform the regression analysis based on the metadata collected. Beta diversity analyses using Non-Metric Multidimensional Scaling (nMDS) ordinations of Bray Curtis were done using distance metrics computed from the rarefied OTU table and the metadata. We used nMDS ordination, achieved by the metaMDS wrapper function as well as boxplots, which were generated using the vegan package [27]. The ordination was applied such that the data were scaled down to two dimensions. The heatmap showing the taxa that significantly differed in abundance between depths (*p* < 0.05) was built using the *heatmap.3* function in R [28].

## 3. Results and Discussion

Puerto Rico is a small island of 100 by 35 miles. Therefore, by collecting samples along all four sides of the quasi rectangle-shaped island (Figure 1), we were able to get a representative coverage of the bacterioplankton community diversity around the island. The 10 collected samples resulted in an average of 108,095 ± 51,855 raw reads. Due to the variability inherent to water sampling, and the fact that current NGS technologies such as Illumina, have higher error rates than traditional Sanger sequencing (Ratan 2013), we decided to perform a conservative data analyses using only OTUs corresponding to the 100% core microbiome at different categories. The number of sequences used in the analyses was an average of 29,324 ± 10,149 reads (Table 1). These sequences yielded ~2309 ± 221 OTUs.

A total of 32 phyla were present in the 11 sampling sites including one unclassified bacterial and two archaeal phyla. The most dominant phyla across all 11 samples were the Proteobacteria, Bacteroidetes, and Planctomycetes, while the least dominant taxonomic groups were the NKB19, Tenericutes, OP3, Lentisphaerae, and SAR406 (Table S1). A total of 293 Family and/or Genus level-OTUs were found, and the top dominant taxa were the Piscirickettsiaceae, Rhodobacteraceae (*Rhodovulum*), and *Vibrio* (Table S2), which correspond to the core microbiome since they were overrepresented in almost all of the samples. The least dominant taxa at the family and genus levels were the Chthoniobacteraceae, Pelagibacteraceae (SAR11), *Roseovarius,* or *Reinekea* (Table S2). The low detection of ubiquitous Pelagibacteraceae (SAR11) (<0.5%) is notable and was not expected. This is probably associated to the PCR bias of the V4 primer used in this survey. In the GOS dataset, 30.9% of the total 16S rDNA reads retrieved from the shotgun metagenomic dataset belonged to the SAR1 [29], whereas only 0.5–1.45% of the 16S rDNA amplicons reads in the BR (Benham Rise, Philippines, Western Pacific Ocean) dataset belonged to this group [30]. The shotgun metagenomics of the same marine samples revealed that members of SAR11 represent more than 28% of the total prokaryote abundance, meanwhile 16S rDNA amplicons revealed that these ubiquitous group represented less than 2.5% of the total prokaryotic community [31]. The primer set 515F-806R [32] used in this study, has been reported for low bending efficiency of the reverse primer for the SAR11 cluster [33]. Here, we can assume that the underrepresentation of the SAR11 taxa in these pelagic samples is likely due to the inherent PCR primer bias.

**Figure 2.**
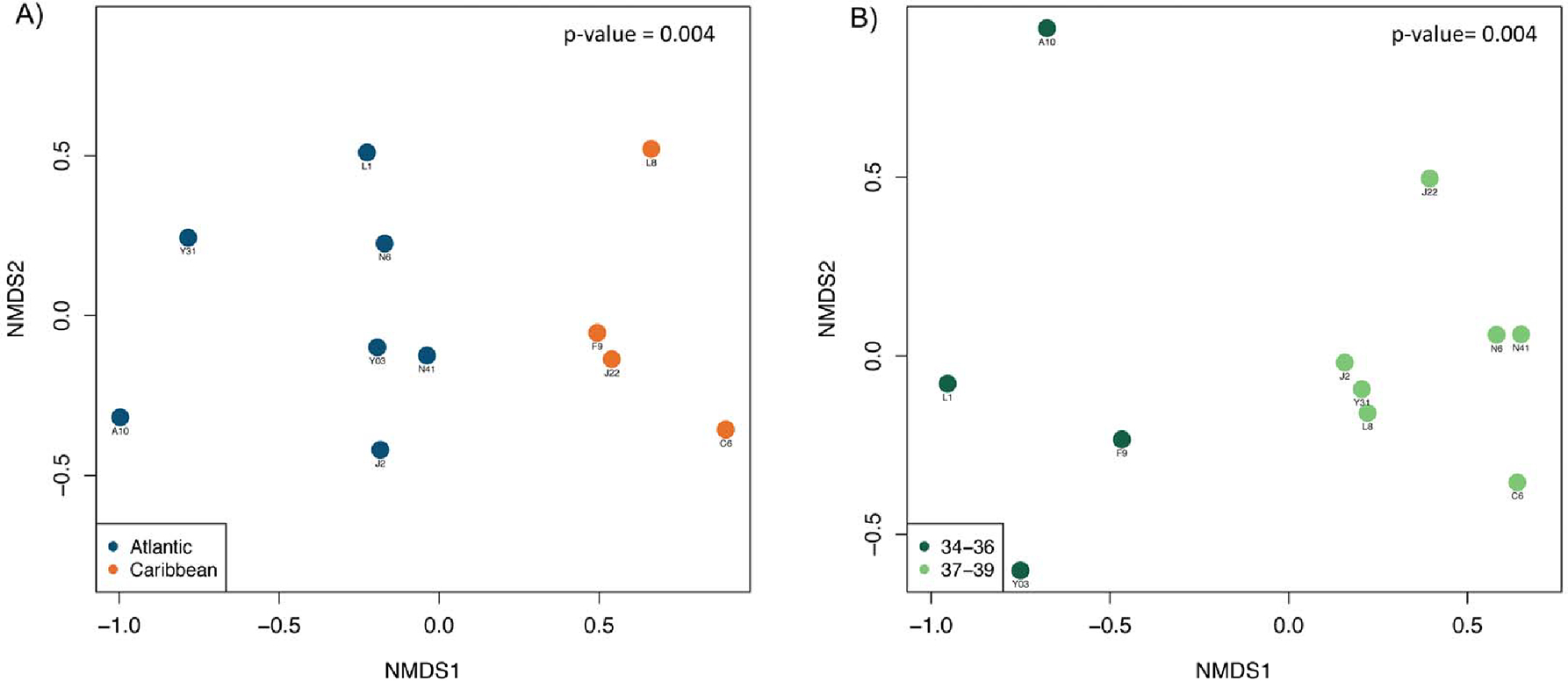
Non-metric multidimensional scaling (NMS) ordination showing the level of dissimilarity of microbial community composition between the geographical area (**A**) and salinity (**B**), as explained by NMS axes 1 (NMDS1) and 2 (NMDS2).

**Figure 3.**
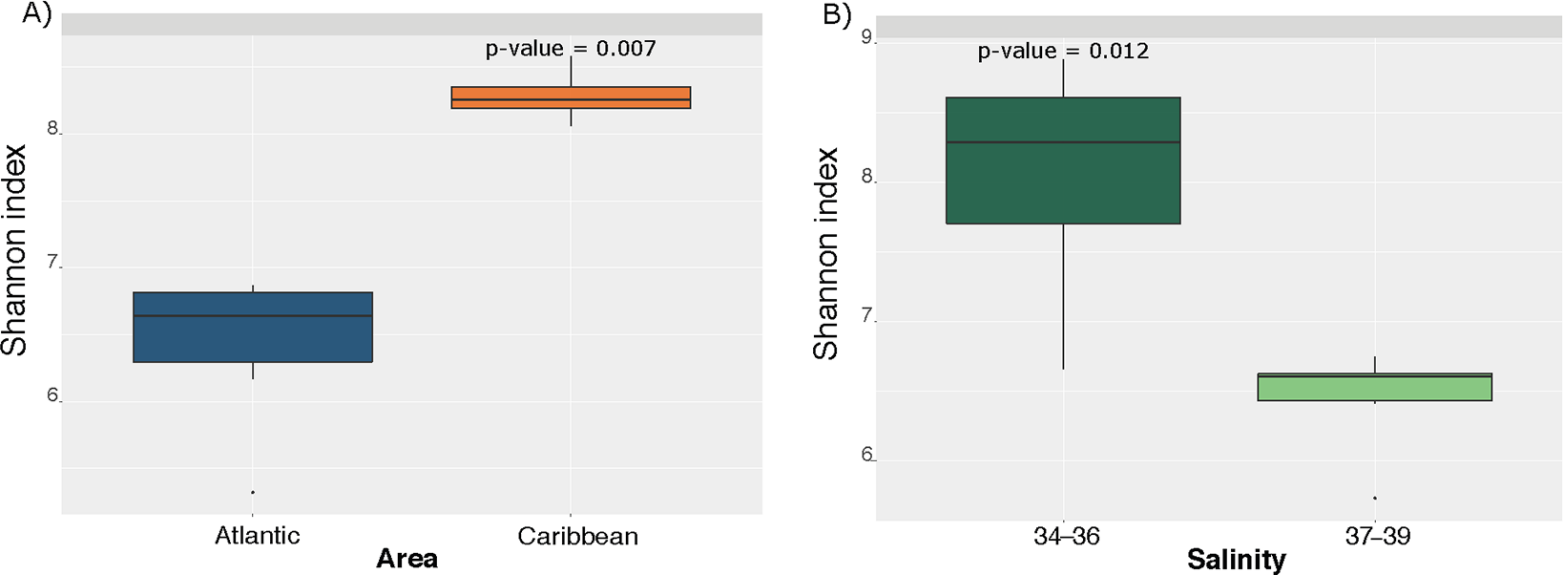
Box plots showing the alpha diversity measures (Shannon index) along the geographical area (**A**) and salinity (**B**).

We found no significant differences in the environmental parameters between sampling sites (dissolved oxygen, pH, temperature), nor between the four cardinal areas, as well as between the sediment and water (*p* > 0.05). However, significant differences in the relative abundance of certain taxa were found between the Atlantic and Caribbean sides of the island. Indeed, we found a clear separation on the beta diversity plot, between the Caribbean and the Atlantic areas (*p*-value = 0.004) (Figure 2A). Interestingly, communities were also separated according to salinity (Figure 2B) (*p*-value = 0.004). Alpha diversity (species diversity) based on the Shannon index, was found to be significantly higher in the Caribbean sampling sites compared to the Atlantic Areas (Figure 3A; *p*-value = 0.007). A significant value was taking into account that the Caribbean area is only composed of four samples, while the Atlantic area is composed of six samples. Similarly, diversity was found to be significantly higher in lower salinity samples 34–36 ppt, compared to those areas with a higher salinity 37–39 ppt (*p*-value = 0.012—Figure 3B). The 34–36 ppt range is made up of four samples and the 37–39 ppt range is composed of six samples. This result suggests that, salinity has a very high impact on microbial community along the littoral coast of Puerto Rico. This result is consistent with previous studies that showed that the species richness and evenness declined as salinity increased along an estuarine salinity gradient [34].

**Figure 4.**
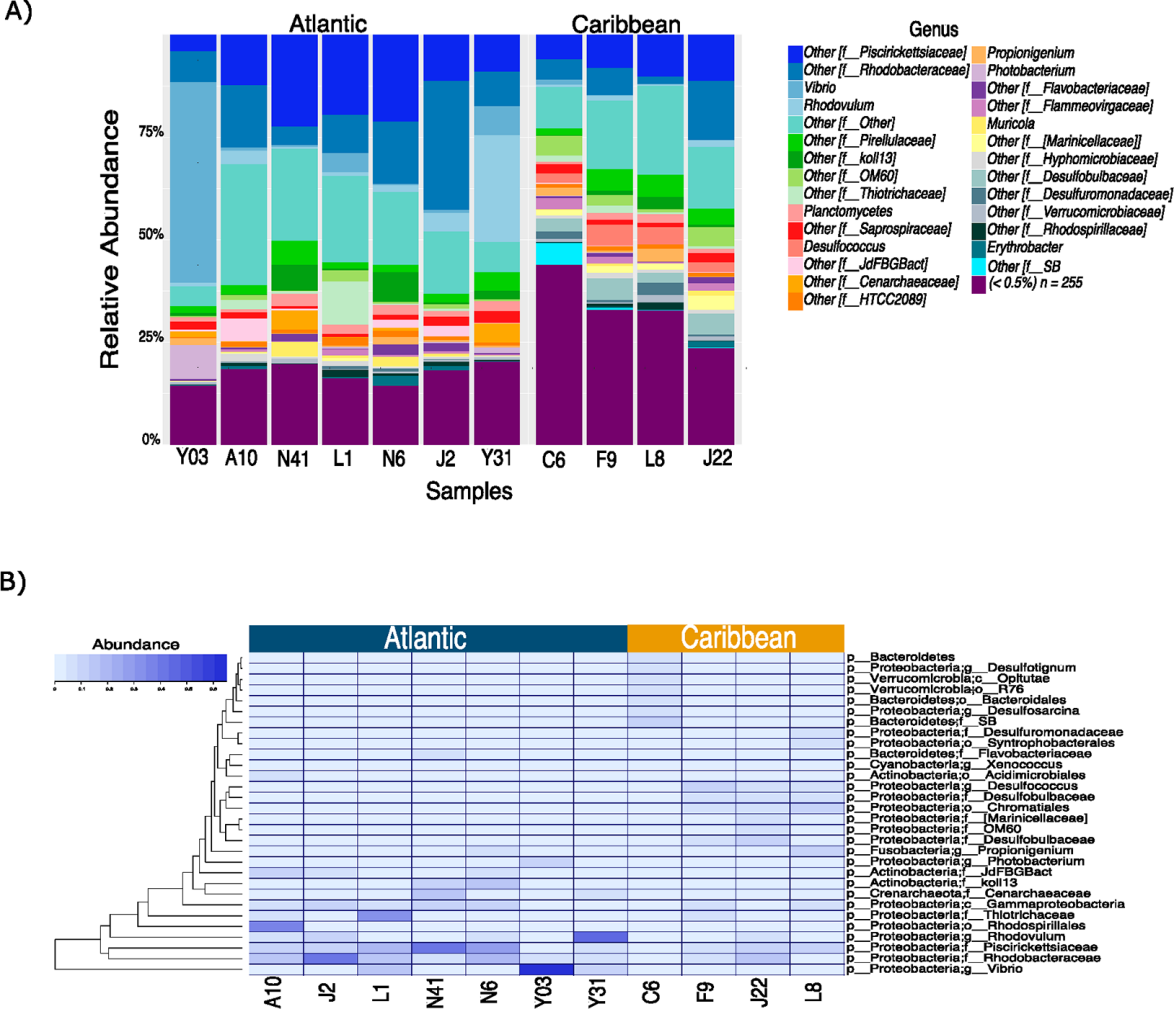
Taxonomic assignment according to the geographical areas. Bar plot showing the relative abundance of different taxa (mostly at the genus and family levels) in the 11 samples (**A**). Heatmap showing the most significant OTUs by area (*p*-value < 0.05) (**B**).

**Figure 5.**
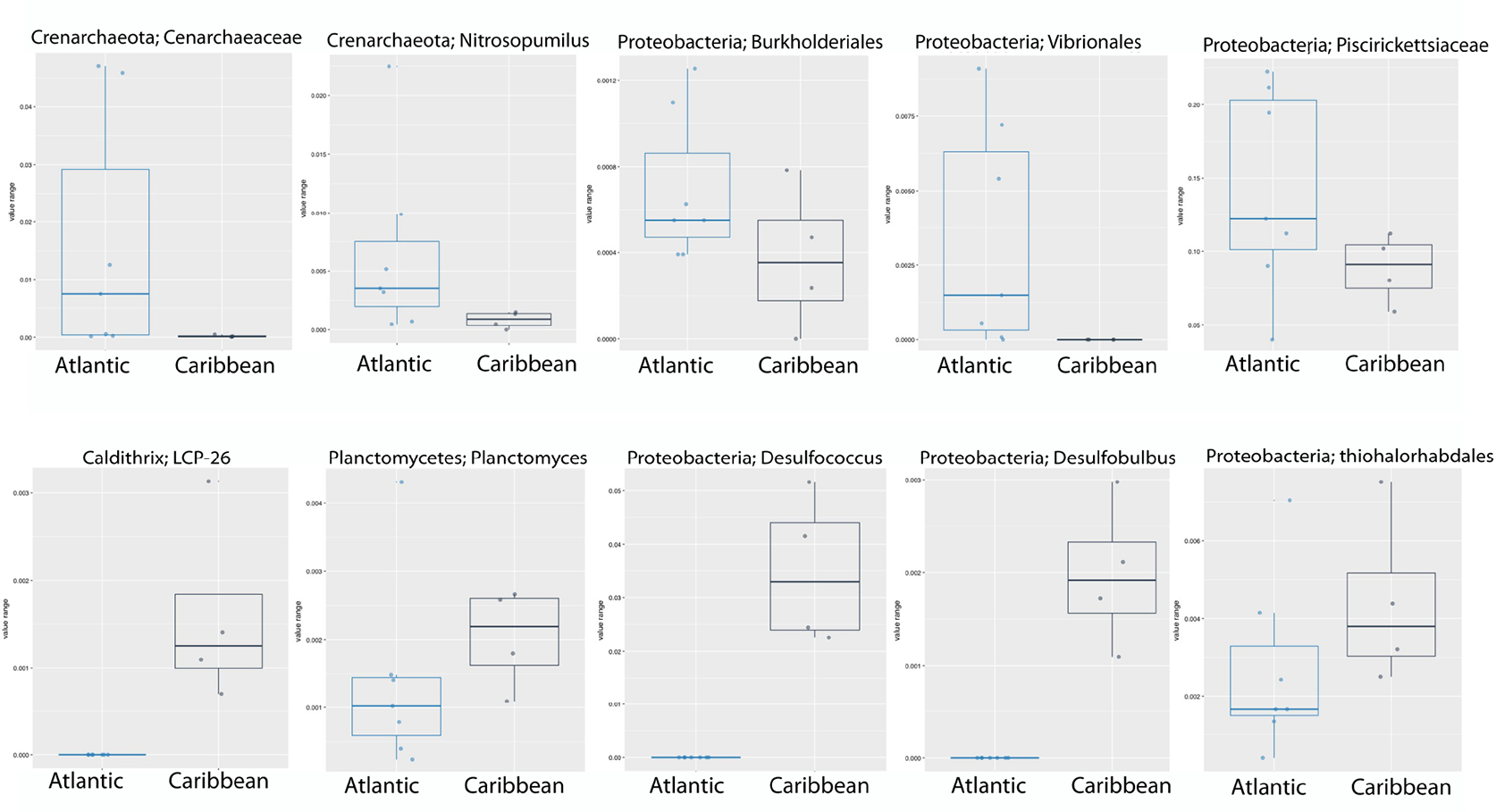
Boxplots of significantly different genus-level OTUs in the Atlantic and Caribbean sites.

The main differences between the microbial communities of the Atlantic and Caribbean include a higher abundance of *Verrucomicrobia* and *Bacteroidetes* in the Caribbean, while *Preoteobacteria*, *Actinobacteria,* and *Crenarchaeota* were more abundant in the Atlantic sites (Figure S1). At the family and genus levels, we found the Atlantic areas to be significantly more abundant in V*ibrio, Rhodobacteraceae, Rhodovulum,* and *Pisciricketsiaceae*, while *Chromatiales*, *Desulfococcus*, *Desulfobacteraceae* (sulfate reducing bacteria), *Flameovirgaceae* or *Alteromonadales*, *Pirellulaceae,* and other *Planctomycetes* were more abundant in the Caribbean (Figure 4A,B and Figure 5). Sulfate reducing bacterial consortia such as *Desulfococcus* and *Desulfobulbus* have also been found to be abundant in the Caribbean sites (Figure 5), and were found mostly associated with coral systems [2,35,36]. The increase in sulfate-reducing microorganisms in the cycling of elements in Caribbean sites, is thermodynamically favored in relation to, for example, methanogenesis, and could be related to a decrease in the methane flux in the Caribbean sites. An increase in halophilic taxa such as genus *Thiohalorhabdus* is also shown in the Caribbean sites.

Some of the bacterial taxa which were found in our survey include a diversity of aerobic generalistic hydrocarbon degraders, which can utilize hydrocarbons and non-hydrocarbon substrates as a source of carbon and energy such as Pisciricketsiacea*e* (Figures 4B and 5). These findings supported previous ones on microbial taxa found in the Deepwater Horizon oil spill [37]. Planctomycetes, abundant in the Caribbean, are reported worldwide in marine water samples and are especially known to be associated with microalgae [38]. This association with marine bacterioplankton and algae is of considerable interest as the bacteria may play an active role in controlling harmful algal blooms in oceans. Moreover, the increase of this taxa in the Caribbean, which maybe associated to ammonia is oxidized by nitrate to nitrogen gas, yielding energy [39].

Therefore, the Caribbean is a good model for these expected changes due to lower salinity levels and elevated silica [40]. In addition, understanding the microbial ecology of the different marine ecosystems is essential for our ability to assess the importance of biogeochemical cycles-climate feedbacks in the climate change era.

## 4. Conclusions

Microbes regulate the health and dynamics of most ecosystems. Therefore, it is vital to continuously monitor not only terrestrial but also aquatic ecosystems in order to develop quick responses to disturbances. Our 16S rRNA survey of samples collected across the island of Puerto Rico revealed that the geographical location and salinity were the main drivers of microbial structure in pelagic water. Our findings suggest that microbial community diversity across the island of Puerto Rico changes depending on the geographic location of either the Atlantic and the Caribbean with an increase in sulfate reducers and ammonia oxidizers. Remarkably, we found that water salinity is the other main driver of the bacterial assemblages in the pelagic water across this Caribbean Island. Sea surface salinity is an essential variable of climate change, which plays a fundamental role in ocean circulation, water circle, and climate. This result suggests that marine microbial communities are very sensitive to very small changes in sea surface salinity. It is important to highlight the limitation of this survey and some other microbiome analysis, as these rely on PCR amplification of 16S rRNA and some key taxa may fail to be detected due to the inherent primer bias and sensitivity. Furthermore, a high diversity of the metabolic capacities of the microbial communities could not be assessed using the 16S rRNA approach. Further studies with more extensive sampling around the coastal water of this Caribbean region with the use of shotgun metagenomics will provide more insight regarding the response of the microbial communities and functions to environmental changes in this local marine ecosystem.

As demonstrated before, the role played by anthropogenic and natural impacts can be significant as well [41]. We found the Caribbean Sea samples to be an interesting model to study climate change due to the relative abundance of sulfate reducing bacteria, previously associated to the demise of coral ecosystems [42]. Nonetheless, the few numbers of samples suggest that the microbiota around the island of Puerto Rico warrant further time-scaled investigations. The island is climatically characterized by two different seasons, the rainy season that goes from July through November and the dry season from December until June. Heavy precipitations or drought are known to alter the salinity or pH of water bodies, thereby significantly changing the local microbial community [43]. Understanding how the natural water microbiota assemblages change in the Caribbean, may shed light on how global phytoplankton will cope with a changing climate. Although they have been reported to contribute very little to global warming, island nations seem to be especially vulnerable to the impacts of climate change [44].

One of the most alarming effects is the sea level rise directly caused by the expansion of seawater as it warms. These entail among others, marine species dying out or changing their metabolism and range. Such loss in biodiversity is proven to be detrimental to the biogeochemical cycling of nutrients including atmospheric gas production and carbon sequestration by microbes in the oceans. The balance of all these cycles and compounds controls the dynamics of all ocean biomes. Therefore, it is paramount to continue looking at microorganisms in order to get a better understanding of global change in the quest of finding solutions to unprecedented and undesirable changes.

## Supplementary Material

Supplementary Table 1. Phyla-level taxa per each sample

Supplementary Figure 1. Significant changes at the phyla-level for bacteria among Atlantic and Caribbean collected samples

Supplementary Table 2. genus-level taxa per each sample

## Figures and Tables

**Table 1.** Description of the 11 samples based on the sampling area, environment, and number of sequences and operational taxonomic units (OTUs).

Sample ID	Site	Coordinates	Sample	Region	Number of Sequences	Number of OTUs
C6	Cabo Rojo	Lat: 18° 05’ 15” N, Lon: 067° 08’ 49” W	Water	Caribbean	21,493	2268
F9	Yabucoa	Lat: 18° 03’ 01” N, Lon: 066° 52’ 45” W			25,022	2716
A10	Humacao	Lat: 18° 09’ 18” N, Lon: 065° 49’ 13” W		Atlantic	25,366	2162
L1	Fajardo	Lat: 18° 19’ 32” N, Lon: 065° 39’ 08” W			22,788	2461
J2	San Juan	Lat: 18° 27’ 55” N, Lon: 066° 06’ 20” W			25,583	2618
Y03	Arecibo	Lat: 18° 20’ 47” N, Lon: 066° 45’ 10” W			57,110	2268
L8	Salinas	Lat: 17° 58’ 39” N, Lon: 066° 17’ 452” W	Sediment	Caribbean	29,605	2027
J22	Ponce	Lat: 18° 00’ 39” N, Lon: 066° 36’ 50” W			27,670	2239
N6	Mayagüez	Lat: 18° 12’ 04” N, Lon: 067° 08’ 42” W		Atlantic	29,066	2231
N41	Aguadilla	Lat: 18° 25’ 38” N, Lon: 067° 09’ 14” W			29,537	2102
Y31	Arecibo	Lat: 18° 20’ 47” N, Lon: 066° 45’ 10” W			31,134	1052
