# Supplementary figures and images for: Divergent Microbiota Dynamics along the Coastal Marine Ecosystem of Puerto Rico
Source: Microbiol Res (Pavia). Author manuscript; Available in PMC 2024 Aug 22. (PMC11340205; doi:10.3390/microbiolres11020009)

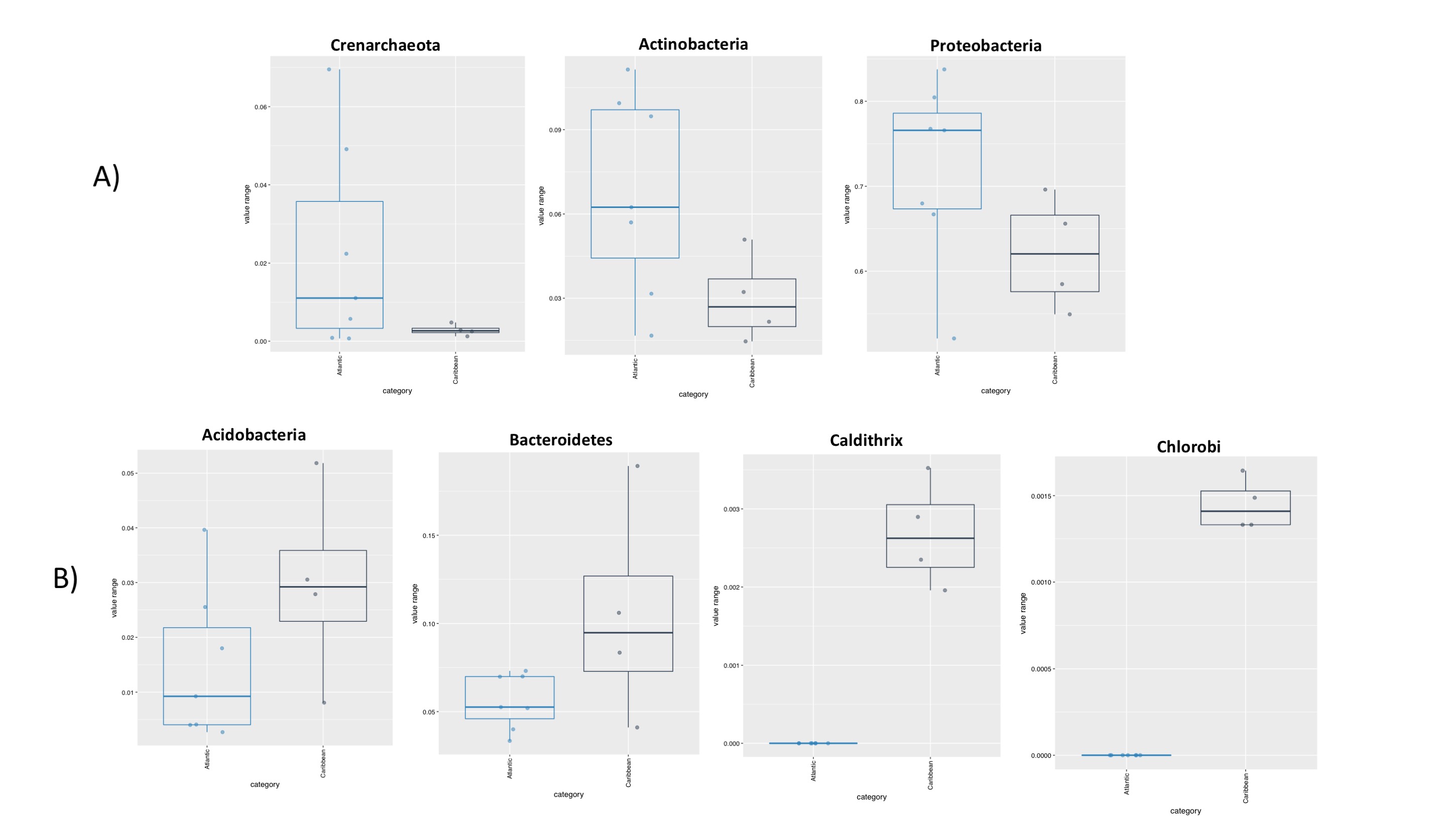

Supplement: Supplementary Figure 1. Significant changes at the phyla-level for bacteria among Atlantic and Caribbean collected samples [file NIHMS2008943-supplement-Supplementary_Figure_1__Significant_changes_at_the_phyla-level_for_bacteria_among_Atlantic_and_Caribbean_collected_samples.jpeg]
